# Impact of a Microbial Cocktail Used as a Starter Culture on Cocoa Fermentation and Chocolate Flavor

**DOI:** 10.3390/molecules22050766

**Published:** 2017-05-09

**Authors:** Igor Magalhães da Veiga Moreira, Leonardo de Figueiredo Vilela, Maria Gabriela da Cruz Pedroso Miguel, Cledir Santos, Nelson Lima, Rosane Freitas Schwan

**Affiliations:** 1 Post-Graduate Program in Food Science, Federal University of Lavras, Lavras 37.200-000, Minas Gerais, Brazil; moreira.igor@gmail.com; 2 Post-Graduate Program in Agricultural Microbiology, Federal University of Lavras, Lavras 37.200-000, Minas Gerais, Brazil; leo.biomed@gmail.com (L.d.F.V.); mgcpmiguel@gmail.com (M.G.d.C.P.M.); rschwan@dbi.ufla.br (R.F.S.); 3 Department of Chemical Sciences and Natural Resources, Centro de Excelencia en Investigación Biotecnológica Aplicada al Medio Ambiente (CIBAMA), Scientific and Technological Bioresource Nucleus (BIOREN), Universidad de La Frontera, Temuco 4811-230, Chile; 4 CEB-Centre of Biological Engineering, Micoteca da Universidade do Minho, University of Minho, 4710-057 Braga, Portugal; nelson@ie.uminho.pt

**Keywords:** chocolate quality, GC-MS, sensory analysis, culture-independent analysis, starter culture

## Abstract

Chocolate production suffered a vast impact with the emergence of the “witches’ broom” disease in cocoa plants. To recover cocoa production, many disease-resistant hybrid plants have been developed. However, some different cocoa hybrids produce cocoa beans that generate chocolate with variable quality. Fermentation of cocoa beans is a microbiological process that can be applied for the production of chocolate flavor precursors, leading to overcoming the problem of variable chocolate quality. The aim of this work was to use a cocktail of microorganisms as a starter culture on the fermentation of the ripe cocoa pods from PH15 cocoa hybrid, and evaluate its influence on the microbial communities present on the fermentative process on the compounds involved during the fermentation, and to perform the chocolate sensorial characterization. According to the results obtained, different volatile compounds were identified in fermented beans and in the chocolate produced. Bitterness was the dominant taste found in non-inoculated chocolate, while chocolate made with inoculated beans showed bitter, sweet, and cocoa tastes. 2,3-Butanediol and 2,3-dimethylpyrazine were considered as volatile compounds making the difference on the flavor of both chocolates. *Saccharomyces cerevisiae* UFLA CCMA 0200, *Lactobacillus plantarum* CCMA 0238, and *Acetobacter pasteurianus* CCMA 0241 are proposed as starter cultures for cocoa fermentation.

## 1. Introduction

The cocoa (*Theobroma cacao* L.) supply chain for the production of chocolate is complex. It involves several post-harvest steps, which can determine the quality of the final product. In Brazil, cocoa production suffered a vast impact with the emergence of “witches’ broom” disease [1,2]. In order to recover the cocoa production, many disease-resistant hybrid plants, such as PH9, PH15, PH16, PS1030, PS1319, CCN51, CEPEC2002, CEPEC2004, and FA13, have been developed [3,4].

As a matter of consequence, different cocoa hybrids generate cocoa beans that produce chocolate with variable quality [5,6,7]. In this context, PH15 hybrid has great relevance due to high-productivity, adaptation, and resistance to some diseases, such as “witches’ broom” and ceratocystis wilt [8,9,10].

The fermentation of cocoa beans is a microbiological process with enzymatic activity and the development of chocolate flavor precursors [11,12,13]. This traditional process is spontaneous and uncontrolled. After opening of the cocoa pods, the beans are transferred to the area of fermentation and placed in heap or fermentation boxes. These methods are the most commonly used among the cocoa producer countries [14,15].

Yeasts, lactic acid bacteria (LAB), and acetic acid bacteria (AAB) are the main microbial communities involved during cocoa fermentation. Yeast species are reported as the primary colonizers of cocoa fermentation. *Saccharomyces*, *Hanseniaspora* (anamorph *Kloeckera*), and *Pichia* are the prevalent genera found in cocoa fermentation in different countries. *Saccharomyces cerevisiae* is particularly the most reported species in many fermentations [16,17,18,19,20].

Simultaneously with the yeast growth, LAB colonize the cocoa mass and degrade the pulp’s glucose into lactic acid and assimilate the citric acid also present in the pulp. Several studies concerning the microbial fermentation reported two LAB species as the most prevalent in this process: *Lactobacillus plantarum* and *Lactobacillus fermentum* [19,20,21].

Yeast populations, which are responsible for the ethanol production, decline together with the LAB populations. AAB dominates the process and are responsible to the exothermic reaction of ethanol conversion into acetic acid. *Acetobacter pasteurianus* is the most frequent species of AAB found in cocoa fermentation, but other species, such as *Acetobacter aceti*, *Acetobacter ghanensis*, *Acetobacter fabarum*, *Gluconobacter oxydans*, and *Gluconobacter xylinus*, have also been reported in the literature [16,17,20,22].

Species of *Bacillus* (e.g., *Bacillus subtilis*, *Bacillus megaterium*, and *Bacillus flexus*) may also grow during fermentation and can affect bean quality and cocoa flavor [16,17,23].

Different compounds, such as alcohols (e.g., 2-methyl-1-propanol, 2-phenylethanol, methanol), aldehydes (e.g., acetaldehyde, benzaldehyde), ketones (e.g., 2-pentanone, phenylmethyl ketone), esters (e.g., ethyl acetate, 2-phenylethyl acetate), and carboxylic acids (e.g., butanoic acid, nonanoic acid), are produced during fermentation, affecting the final flavor character in chocolate [13,17,24,25,26].

The aim of this work was to use a cocktail of microorganisms as a starter culture on the fermentation of the ripe cocoa pods from PH15 cocoa hybrid, and evaluate its influence on the microbial communities present on the fermentative process, on both the volatile and non-volatile compounds produced during the fermentation, and to perform the chocolate sensorial characterization.

## 2. Results

### 2.1. Culture-Independent Analysis with PCR–DGGE

**Figure 1:**
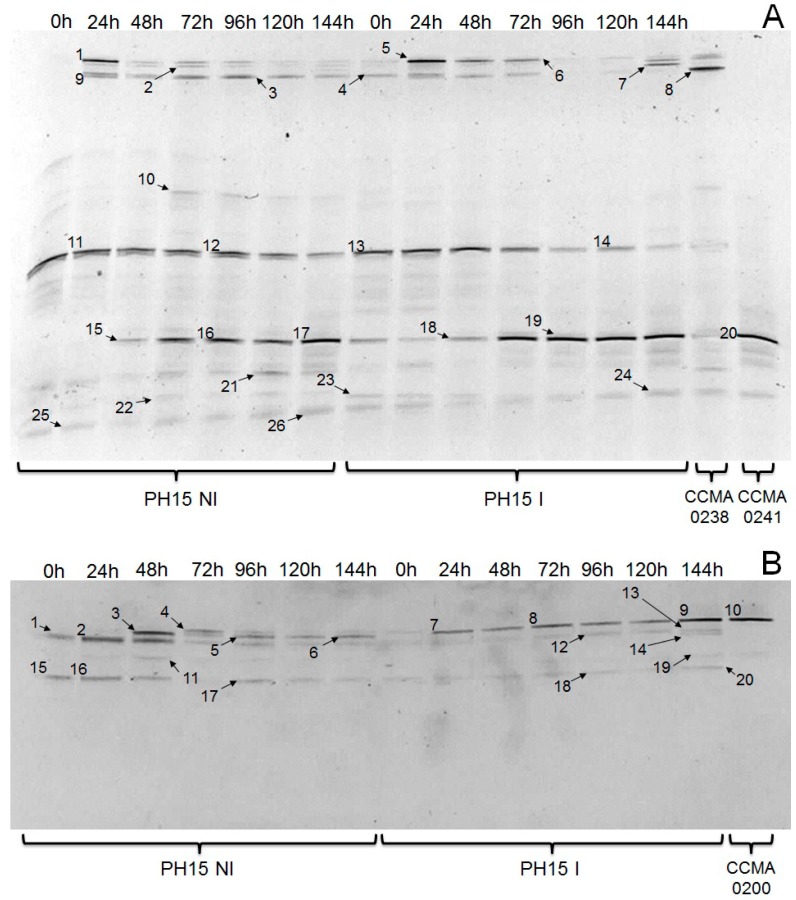
Changes in prokaryote (**A**) and eukaryote (**B**) communities during fermentation of the PH15 cocoa hybrid without (PH15 NI) and with (PH15 I) inoculation, and fingerprints of the starter cultures (CCMA 0238, CCMA 0241, and CCMA 0200). The identities of the bands are presented in Table 1. Bands marked with numbers were excised, re-amplified, and sequenced.

Analyses of the microbial communities on both inoculated and non-inoculated samples of the PH15 cocoa hybrid were performed by Polymerase Chain Reaction Denaturing Gradient Gel Electrophoresis (PCR–DGGE) for prokaryote (Figure 1A) and eukaryote (Figure 1B) microorganisms. Identification of DGGE bands are shown in Table 1.

The bacterial and yeast communities changed according to different fermentation processes (inoculated or non-inoculated). The bacterial species *Gluconobacter oxydans* (bands 22–24), *Lactobacillus plantarum* (bands 3, 4, 8, and 9), uncultured bacterium (bands 11–15), *Acetobacter pasteurianus* (bands 15–20), and *Fructobacillus pseudoficulneus* (bands 1, 5, and 6) were detected for both inoculated and non-inoculated fermentations.

The species *Leuconostoc* sp. (band 2), *Zymomonas mobilis* (Band 10), *Acetobacter* sp. (Bands 21), *Bacillus* sp. (Bands 25 and 26) were detected in the non-inoculated fermentation. The *Lactobacillus helveticus* (Band 7) was only detected in inoculated fermentation. The yeast species *Saccharomyces cerevisiae* (Bands 3 and 5–10) and *Pichia kluyveri* (Bands 15–18 and 20) were detected for both fermentations. *Saccharomyces* sp. (Band 4), *Hanseniaspora uvarum* (Bands 1 and 2), and *Theobroma cacao* (Band 11) were only detected in the non-inoculated fermentation: this was possible because universal eukaryote primers were used. In the inoculated fermentation *Rhodotorula mucilaginosa* (Bands 12 and 13), *Trichosporon asahii* (Band 19), and *Lentinula edodes* (Band 14) were detected.

### 2.2. Chemical Changes During Fermentation

Temperature and pH values were measured during 120 h of fermentation. In the fermentation of PH15 NI the temperature varied from 30.7 °C at 0 h (with the maximum of 49.1 °C at 96 h) to 47.9 °C at 120 h. On the other hand, in the fermentation of PH15 I, the temperature varied from 26.62 °C at 0 h (with the maximum of 50.06 °C at 72 h) to 49.88 °C at 120 h. The pH value outside the bean (pulp) varied during fermentation. The pH value of PH15 NI ranged from 3.27 to 4.45, and the fermentation PH15 I pH value ranged from 3.27 to 4.81.

#### 2.2.1. Sugar Consumption and Metabolite Production

**Figure 2:**
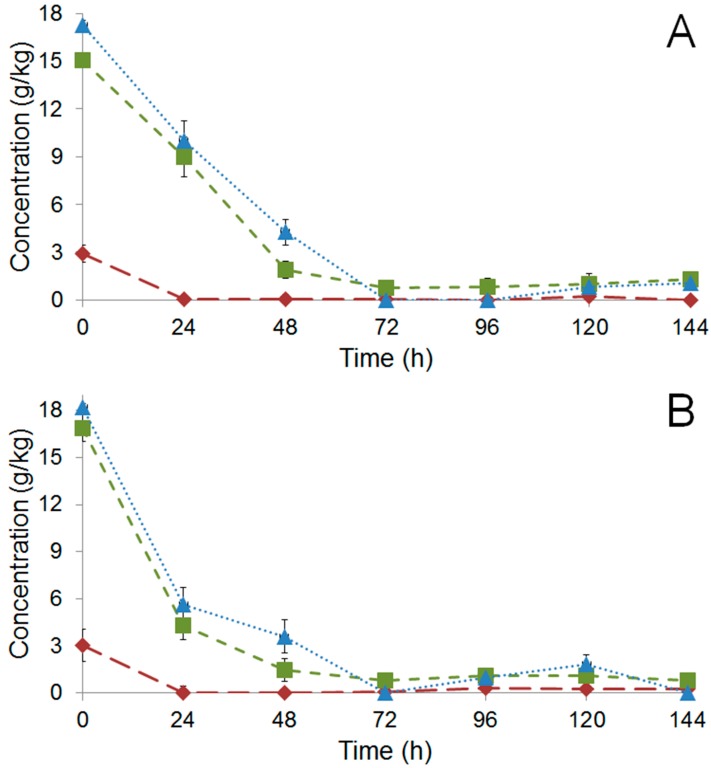
Course of glucose (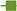), fructose (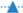), and citric acid (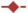) during fermentation of PH15 NI (**A**) and PH15 I (**B**).

During the six days of fermentation the concentrations of glucose, fructose, and citric acid were evaluated in the pulp, and the results are shown in Figure 2. Citric acid was fully metabolized at 24 h of fermentation in both assays (Figure 2A,B). In both fermentations, glucose and fructose were completely consumed at 72 h. After 24 h of inoculated fermentation the PH15 sample showed greater sugar consumption compared to the non-inoculated one, but in the following hours, both inoculated and non-inoculated fermentations presented the same profile of sugar consumption (Figure 2A,B).

**Figure 3:**
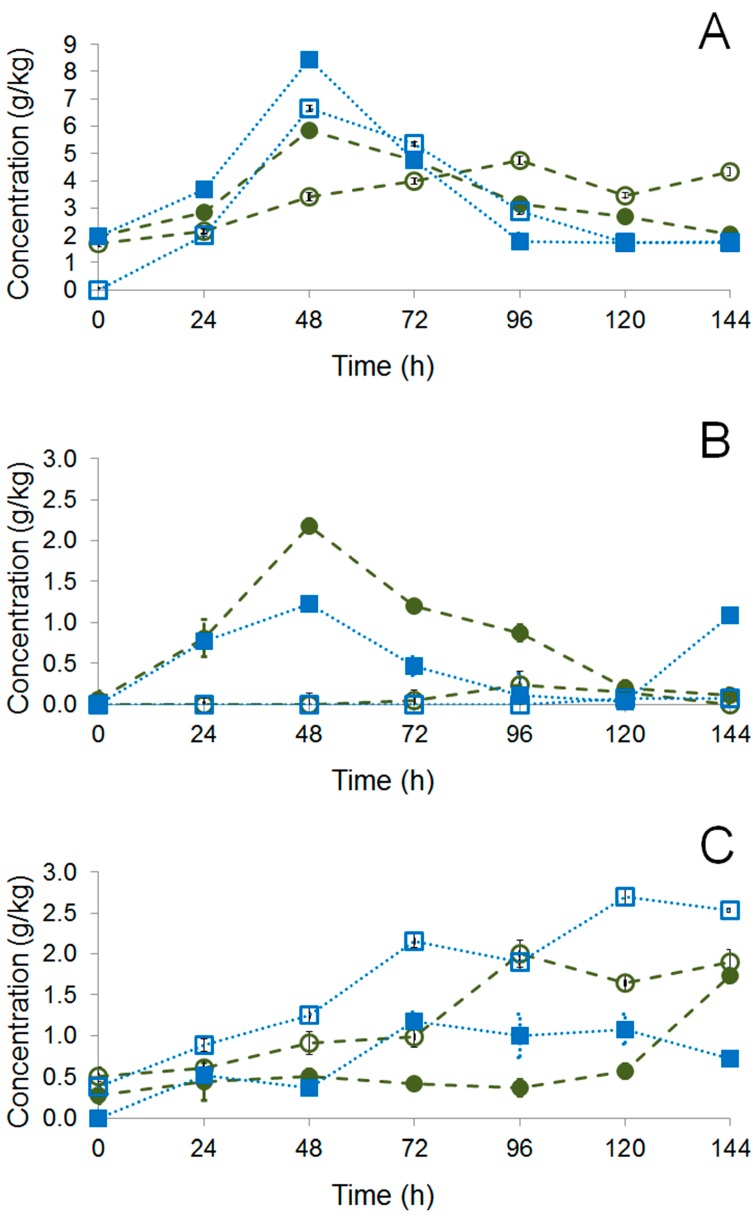
Detection by HPLC of ethanol (**A**); lactic acid (**B**); and acetic acid (**C**) during 144 h of fermentation of PH15 NI (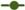) and PH15 I (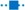); full symbols correspond to metabolites detected in the pulp while open symbols to those detected inside the beans.

Ethanol, lactic acid, and acetic acid were evaluated in the pulp and inside the beans, and are shown in Figure 3. The inoculated fermentation of PH15 showed the maximum value of ethanol in the pulp (8.44 g/kg at 48 h), and this compound inside the beans also reached higher values (6.65 g/kg at 48 h) when compared to the control fermentation (PH15 NI) (Figure 3A). However, at the end of the fermentation, the ethanol concentration was higher (4.34 g/kg) inside the beans in the non-inoculated fermentation.

The microbial inoculation accelerated the sugar consumption in the first 24 h of cocoa fermentation, whereas the ethanol production was accelerated in the first 48 h of fermentation (Figure 2B and Figure 3A). The pulp of PH15 I showed the highest acetic acid concentration between 48 h and 120 h of cocoa fermentation, and the lowest concentration at the end of the fermentation (144 h, Figure 3C). In contrast, PH15 NI showed the highest acetic acid concentration in the residual pulp at the end of the fermentation (144 h, Figure 3C). Overall, the concentration of acetic acid was higher in the fermentation of PH15 I (Figure 3C).

After 144 h, the pulp of PH15 I showed greater concentration of lactic acid than the pulp of PH15 NI (Figure 3B). In both PH15 I and PH15 NI samples there was no lactic acid penetration in the cotyledon. Acetic acid was higher inside the beans in PH15 I at later stages, at 120 h (2.70 g/kg), and at 144 h (2.53 g/kg).

#### 2.2.2. Volatile Compounds

A total of 37 volatile compounds were detected by Gas Chromatography Mass Spectrometry (GC–MS) at the beginning (0 h), and a total of 38 volatile compounds at the end (144 h), of the non-inoculated fermentation. While in the inoculated fermentation, a total of 37 volatile compounds were detected at the beginning (0 h) and a total of 34 volatile compounds at the end (144 h), as presented in the Table 2.

**Figure 4:**
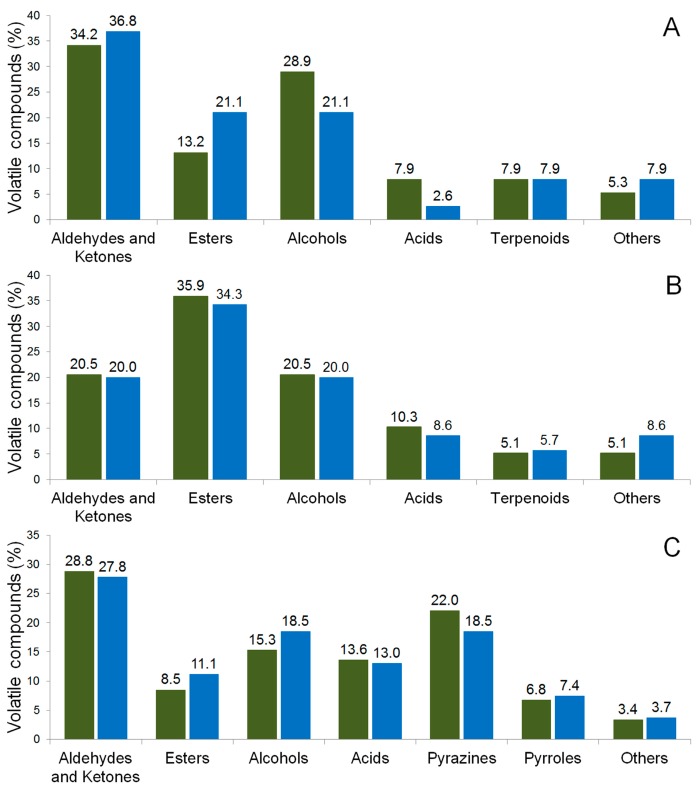
Profile of volatile compounds identified by HS–SPME GC–MS during non–inoculated fermentation (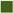 PH15 NI), inoculated fermentation (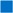 PH15 I), and in the chocolate samples. Fermentation times: 0 h (**A**) and 144 h (**B**). Chocolate samples (**C**). Total amount of compounds: PH15 SI 0 h (37), PH15 SI 144 h (38), PH15 SI Ch (58), PH15 I 0 h (37), PH15 I 144 h (34), and PH15 I Ch (54).

Both non-inoculated and inoculated hybrid PH15 (0 h and 144 h) showed the following identified compounds: aldehydes and ketones, acids, alcohols, esters, terpenoids, and furans. Aldehydes and ketones occurred at the beginning (0 h) and esters occurred at the end (144 h). In both fermentations, the most important groups of volatile compounds were detected (Figure 4).

Chocolate samples of PH15 I and PH15 NI presented 58 and 54 volatiles compounds, respectively (Table 2 and Figure 4). The compounds identified were aldehydes and ketones, acids, alcohols, esters, pyrazines, pyrroles, and furans, and the most important groups detected, in both chocolate samples, were aldehydes and ketones, alcohols, and pyrazines (Figure 4).

### 2.3. Sensorial Analyses of Chocolate

**Figure 5:**
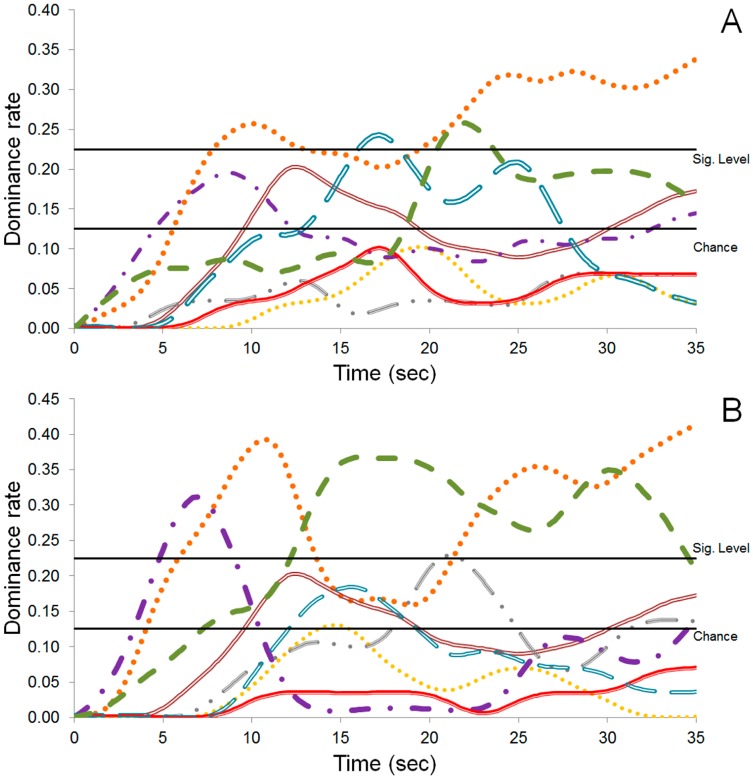
Temporal Dominance Sensory of chocolate produced from cocoa fermented beans PH15 NI (**A**) and PH15 I (**B**). Sensorial attributes: Acid (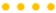), Bitterness (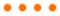), Nutty (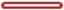), Sweetness (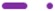), Astringent (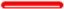), Coffee (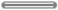), Fruity (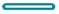), and Cocoa (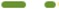).

The chocolate analyses by the Temporal Dominance of Sensations (TDS) technique are shown in Figure 5. The judges noted difference between the two samples of chocolate during the tasting time. The bitterness was the dominant taste in the final time (25–35 s) of PH15 NI Ch (no-inoculated chocolate). However, the fruity and cocoa flavors were significant at 17 and 22 s, respectively (Figure 5A).

The sample PH15 I Ch showed a mixture of sensations, alternating between bitterness, cocoa taste and sweetness (Figure 5B). The bitterness is the more dominant taste in the initial (5 to 10 s) and final time (25 to 35 s), while the cocoa taste was dominant in the intermediate (15 to 20 s) and final time (25 to 35 s). The sweetness taste showed significant levels in the final time (25 to 35 s) (Figure 5B).

## 3. Discussion

In order to evaluate their influence on the fermentation of cocoa beans and on the final sensorial characteristics of produced chocolate, *S. cerevisiae* UFLA CCMA 0200, *Lactobacillus plantarum* CCMA 0238, and *Acetobacter pasteurianus* CCMA 0241 were used as starter cultures for the cocoa PH15 fermentation. The organic compounds and microbial communities involved during the fermentation of non-inoculated and inoculated cocoa were analyzed. Furthermore, the sensorial characterization of chocolate (PH15 I Ch and PH15 NI Ch) produced from the fermented beans was evaluated.

The PCR–DGGE analyses showed that the bacterial and yeast communities were different according to the fermentation process. This may be explained by the use of starter cultures that may have generated changes in the natural microbiota, as shown in Figure 1A,B. Species of LAB and AAB were identified in both fermentations.

The *L. plantarum* and *Fructobacillus pseudoficulneus* species were the bacteria present in both fermentations. However, in PH15 I these species were only identified until in the middle of the fermentation period (72 h), but after this time, they seem not to be present. It is reported that the increase of ethanol concentration during cocoa fermentation inhibited *L. fermentum* growth [15,25]. This could explain the low population rate of *L. plantarum* in PH15 NI.

*Gluconobacter oxydans* and *A. pasteurianus* were AAB species identified in both fermentation processes. In addition, *A. pasteurianus* (Figure 1A—bands 15, 16, 17, 18, 19, and 20) was present at all fermentation times of PH15 I, and this did not happen in the fermentation without inoculum. These species have been described in cocoa bean fermentation in Brazil, Ghana, and Indonesia [3,16,19,20].

The LAB species *Leuconostoc* sp., *Lactobacillus helveticus* were detected in non-inoculated and inoculated fermentations, respectively. The *Zymomonas mobilis*, an ethanol strain producer, *Acetobacter* sp., an AAB species, and *Bacillus* sp. were only detected in non-inoculated fermentation (Figure 1A). The *Bacillus* sp. present in PH15 I fermentation can be explained as the fermentation was not in aseptic conditions. *Bacillus* sp. and filamentous fungi may participate in the spontaneous cocoa bean fermentation process after four or five days of fermentation [14,16,17,20,25].

Fingerprinting based on PCR–DGGE showed that the most common yeast *Saccharomyces cerevisiae* was present during the fermentation in both fermentations. This fact indicated that this yeast may be a promising starter culture used for the cocoa fermentation process. Some works using *S. cerevisiae* as a starter culture have been reported [4,7], and concluded that yeast inoculation accelerated the fermentation process.

The microbial activity and metabolites produced during the cocoa beans’ fermentation leads to an increase of temperature and pH value [19]. Therefore, this may explain the temperature and pH increase in both fermentations, but in PH15 I there was a greater increase.

According to the chemical results, carbohydrates were consumed faster in the inoculated assay (Figure 2B). This is likely due to the higher microorganism population in the inoculated assay than in the control. Further, higher ethanol concentrations (almost two times the concentration detected in the control) were observed in this assay (Figure 3A). However, this was not the case for acetic acid, similar to results previously described elsewhere [4]. However, in inoculated fermentation, acetic acid was detected in the cotyledon at the end of fermentation (Figure 3C). Sucrose was not detected in the fermentation probably because it was hydrolyzed into glucose and fructose when the pods were broken open, as previously described [4].

In addition, to produce primary metabolites, such as ethanol, and lactic and acetic acids during cocoa fermentation, starter cultures also produce a vast array of volatile secondary metabolites, such as higher alcohols, acids, esters, aldehydes, ketones [24,27], and others that could influence cocoa flavor [25].

The most important volatile compound groups detected were esters and alcohols, in both fermentations (Table 2 and Figure 4). These compounds are already described as important in cocoa products [24]. The esters are correlated to fruity notes [28] and the alcohols with flowery and candy notes [29,30], e.g., 2,3-butanediol, 2-heptanol, guaiacol, benzyl alcohol, and phenylethyl alcohol found in this study, being that the latter two compounds were found in all chocolate process stages of both fermentations (PH15 I and PH15 NI) (Table 2) [4].

Acids are generally related to unpleasant odors present in cocoa products [24,30,31]. A total of five compounds were identified, some being related to rancid, sour, or fatty odors. However, some acids detected here may present pleasant odors, e.g., 4-hydroxybutanoic acid and hexanoic acid, with sweetish odors, as shown in Table 2 [4].

In order to investigate the influence of a starter culture on the final product, two chocolates were produced and their sensory analyses were evaluated. The judges noted differences between the two chocolate samples (PH15 I Ch and PH15 NI Ch) during the tasting time. Significant differences were observed as described in Figure 5. Bitter was the dominant taste in PH15 NI Ch (Figure 5A) and, in PH15 I Ch, bitter, sweet, and cocoa tastes were dominant (Figure 5B). These results can be corroborated by the analysis of the volatile compounds in the chocolate samples. The 2,3-butanediol, which gives flavor to cocoa butter (sweet chocolate), and 2,3-dimethylpyrazine, which gives caramel and cocoa flavors, were detected only in PH15 I Ch (Table 2). Therefore, that dominant flavor detected in PH15 I Ch could be related to these compounds, indicating the inoculation influence in the final product.

## 4. Materials and Methods

### 4.1. Fermentation Experiments, Inoculation, and Sampling

The fermentation experiments were conducted at the Vale do Juliana cocoa farm in Igrapiúna, Bahia, Brazil. The ripe cocoa pods from PH15 were harvested during the main crop of 2015 (November).

The cocoa pods were manually opened with a machete, and the beans were immediately transferred to the fermentation house. The fermentation started approximately 4 h after the breaking of the pods and was performed in 0.06 m^3^ wooden boxes [17]. The fermentations were conducted with 100 kg of PH15 cocoa beans. The fermentations were performed using a cocktail of microorganisms (PH15 I) as starter culture containing *S. cerevisiae* UFLA CCMA 0200 (LNF-CA11, LNF Latino America, Bento Gonçalves, Rio Grande do Sul, Brazil), *Lactobacillus plantarum* CCMA 0238 and *Acetobacter pasteurianus* CCMA 0241 at the beginning of the process and without inoculation (PH15 NI-control). These microorganisms were reported in previous studies on cocoa fermentation around the world, mainly in Brazil [3,16,18,20,21]. The pH value and temperature were evaluated during the fermentations.

All of the microbial strains used in the study are preserved at the Culture Collection of Agricultural Microbiology of the Federal University of Lavras (CCMA, Lavras, Minas Gerais, Brazil, WDCM 1083). The *S. cerevisiae* UFLA CCMA 0200, which is commercialized by LNF (CA11), was weighed (as recommended by the manufacturer’s instructions) and mixed in the solution to reach a population of approximately 10^7^ cells/g of cocoa.

The *Lactobacillus plantarum* and *Acetobacter pasteurianus* species were grown in MRS broth (De Man, Rogosa and Sharpe, Merck, Darmstadt, Germany) and YPD broth (10 g/L yeast extract (Merck); 20 g/L peptone (Himedia); 20 g/L dextrose (Merck)), respectively, at 30 °C and 150 rpm, and replicated every 24 h. The cells were recovered by centrifugation (7000 rpm, 10 min) and re-suspended in 1 L of sterile peptone water (1 g/L peptone (Himedia, Mumbai, India)). This solution was spread over the cocoa beans, reaching a concentration of approximately 10^5^ cells/g of cocoa.

The samples were taken every 24 h during 144 h of fermentation and placed in sterile plastic pots. The samples were stored at −20 °C. The fermentations were performed in triplicate [32].

### 4.2. Culture-Independent Microbiological Analysis

#### 4.2.1. DNA Extraction and Polymerase Chain Reaction

The total DNA extraction and PCR reaction from the cocoa pulp were conducted as previously described [3]. Cocoa pulp DNA total was extracted with a QIAamp DNA Mini Kit (Qiagen, Hilden, Germany) in accordance with the manufacturer’s instructions and stored at −20 °C.

The bacterial DNA was amplified with the primers 338fgc (5′-CGC CCG CCG CGC GCG GCG GGC GGG GCG GGG GCA CGG GGG GAC TCC TAC GGG AGG CAG CAG-3′) (the GC clamp is underlined) and 518r (5′-ATT ACCGCG GCTGCT GG-3′). The DNA from the eukaryotic community was amplified with the primers NL1GC (5′-CGC CCG CCG CGC GCG GCG GGC GGG GCG GGGGCA TAT CAA TAA GCG GAG GAA AAG-3′) (the GC clamp is underlined) and LS2 (5′-ATT CCC AAA CAA CTC GAC TC-3′). All reactions were performed in 25 μL containing 0.625 U Taq DNA polymerase (Promega, Madison, WI, USA), 2.5 μL 10 X buffer, 0.1 mMdNTP, 0.2 mM of each primer, 1.5 mM MgCl_2_, and 1 μL of extracted DNA. The amplification was performed as previously described [4]. The amplified products (2 μL) were analyzed by electrophoresis on 1% agarose gels before the DGGE analysis.

#### 4.2.2. PCR–DGGE Analysis

To conduct the DGGE analyses, the PCR products were analyzed using a Bio-Rad DCode universal mutation detection system (Bio-Rad, Richmond, CA, USA). The PCR products were purified, sequenced, and available according to the procedures previously described [3]. Denaturant solutions containing 35–70% (100% denaturant contains 7 M urea and 40% (*v*/*v*) formamide) were used for bacteria, and containing 30–60% for yeast. The electrophoresis was run at 60 °C for 6 h at a constant voltage of 120 V.

### 4.3. Chromatographic Analysis

#### 4.3.1. Sugars, Alcohols, and Organic Acid Extraction and HPLC Analyses

The carbohydrates, alcohols, and organic acids were extracted (from pulp and from the content inside the beans) and analyzed as described in previous work [3]. The analyses were determined by HPLC (Shimadzu, model LC-10Ai, Shimadzu Corp., Kyoto, Japan) equipped with a dual detection system consisting of a Ultraviolet-Visible (UV–Vis) detector (SPD 10Ai) and a refractive index detector (RID-10Ai). The HPLC was operated at 50 °C for acids and detected via UV absorbance (210 nm), while the alcohols and carbohydrates were examined at 30 °C and detected via Refractive Index Detector (RID). The column used for separation was a Shimadzu ion exclusion column (Shim-pack SCR-101H, 7.9 mm × 30 cm, Shimadzu, Kyoto, Japan) with a mobile phase of Perchloric acid (100 mM) at a flow rate of 0.6 mL/min. All samples were analyzed in triplicate.

The chemical compounds used as standards (purity N 99.8%), glucose, fructose, and citric acid, were purchased from Sigma-Aldrich (Saint Louis, MO, USA); acetic acid and ethanol were purchased from Merck (Darmstadt, Germany); and lactic acid was purchased from Fluka Analyticals (Seelze, Germany).

#### 4.3.2. Characterization of Volatile Compounds by Headspace-Solid Phase Microextraction Gas Chromatography-Mass Spectrometry

The volatile compounds from cocoa samples were extracted using the Headspace-Solid Phase Microextraction (HS–SPME) technique, as described in previous research [24], with modifications. Briefly, cocoa samples (2.0 g) from the beginning and end of fermentation (0 h and 144 h) and chocolate samples (2.0 g) were macerated using liquid nitrogen for headspace analysis. A divinylbenzene/carboxen/polydimethylsiloxane (DVB/CAR/PDMS) 50/30 mm SPME fiber (Supelco Co., Bellefonte, PA, USA.) was used to extract volatile constituents from the cocoa and chocolate headspace. The fiber was equilibrated for 15 min at 60 °C and then exposed to the samples (cocoa and chocolate) for 30 min at the same temperature.

The compounds were analyzed using a Shimadzu GC model QP2010 equipped with a mass spectrometry and a capillary column of silica DB-FFAP (25 m × 0.25 mm i.d. × 0.25 mm). The temperature program began with 5 min at 50 °C, followed by a gradient of 50 °C to 190 °C at 3 °C/min; the temperature was then maintained at 190 °C for 10 min. The injector and detector temperatures were maintained at 230 °C.

The carrier gas (He) was used at a flow rate of 1.2 mL/min. Injections were performed by fiber exposition for 5 min. Volatile compounds were identified by comparing the mass spectra of the compounds in the samples with the database of the National Institute of Standards and Technology (NIST library, Gaithersburg, MD, USA) and the retention time with literature data using the n-Alkane index. All samples were examined in duplicate.

### 4.4. Sensory Analysis

After fermentation, the beans were dried in the sun inside drying greenhouses. Thereafter, the dried beans were sent for chocolate production at Sartori and Pedroso Alimentos Ltda. (São Roque, São Paulo, Brazil). Dark chocolate (100 g chocolate bar) was produced (62% liquor, 30% icing sugar, 8% cocoa butter). The molded chocolate was rapped and stored at 4 °C for four weeks before sensory analysis.

For sensory analysis, the chocolate was kept at room temperature (±20 °C) for two hours before the tests. The attributes involved in the TDS analysis were established by the Kelly grid method (“Kelly’s repertory grid method”) [33]. The TDS analysis was performed with 31 selected and trained judges. The judges evaluated differences between the two chocolate samples (PH15 I Ch (from inoculated fermentation) and PH15 NI Ch (from non-inoculated fermentation)) during the tasting time (the analysis time was 35 s, with an addition of delay time 2 s) and the attributes selected were acid, bitterness, nutty, sweetness, astringent, coffee, fruity, and cocoa.

The judges were asked to choose the dominant flavor over the analysis time. The dominant flavor is that perceived with greater clarity and intensity among the other ones [34]. The samples (approximately 2.5 g of chocolate) were presented in plastic cups, coded with a three-digit bar. Crackers and water were provided for palate cleansing. The analysis was performed in triplicate.

In order to calculate the TDS curves for all analyses, the software SensoMaker, version 1.8 was used [35]. Two lines were drawn on graphics: the “chance level” and the “significance level”. The “chance level” is the dominance rate that an attribute can obtain by chance. The “significance level” is the minimum value of this ratio to be considered significant.

### 4.5. Statistical Analyses

Analyses of the variance and the Scott–Knott test were performed with SISVAR 5.1 software (Federal University of Lavras, Department of Statistic, Lavras, MG, Brazil). Differences in values were considered significant when the p value was less than 0.05 (*p* < 0.05).

## 5. Conclusions

The inoculation of microorganisms as a starter culture accelerated the fermentation process. The bacterial and yeast communities were different according to each process (PH15 I and PH15 NI), but the bacteria (*Gluconobacter oxydans*, *Lactobacillus plantarum*, *Acetobacter pasteurianus*, *Fructobacillus pseudoficulneus*) and yeast (*Saccharomyces cerevisiae* and *Pichia kluyveri*) species were found in both processes. Glucose and fructose were consumed faster in the inoculated assay in the first 24 h of fermentation. Different volatile compounds were identified in fermented beans and chocolate produced in the present study. According to the sensory analysis of PH15 I Ch and PH15 NI Ch significant differences on the dominant tastes were observed. The inoculation leads to a chocolate with higher bitter, sweet, and cocoa notes than the chocolate produced by spontaneous fermentation. Bitter was the dominant taste in PH15 NI Ch, whereas bitter, sweet, and cocoa tastes were dominant tastes in PH15 I Ch. These results were corroborated by the analysis of volatile compounds in both chocolate samples. The 2,3-butanediol, which gives flavor to cocoa butter (sweet chocolate), and 2,3-dimethylpyrazine, which gives caramel and cocoa flavors, were detected only in PH15 I Ch. Therefore, that dominant flavor detected in PH15 I Ch was related with these compounds, indicating the inoculation influence in the final product. In this context, the inoculation influenced the fermentation process and the final product. In order to generate a standardized fermentative process and improve the chocolate quality, *Saccharomyces cerevisiae* UFLA CCMA 0200, *L. Plantarum* CCMA 0238, and *A. pasteurianus* CCMA 0241 are, herein, proposed to be used as a cocktail of microorganisms for application in cocoa fermentation.

## Figures and Tables

**Table 1:** Identification of the bands based on Basic Local Alignment Search Tool (BLAST) in comparison with those in GenBank as obtained by PCR–DGGE using universal primers for yeasts and bacteria.

Identification	Bands ^a^	Similarity (%)	Accession Number	Sample Found
**Prokaryotes**				
*Fructobacillus pseudoficulneus*	1, 5, 6	98	AB498052.1	PH15NI, PH15I
*Leuconostoc* sp.	2	97	DQ523491.1	PH15NI
*Lactobacillus plantarum*	3, 4, 8, 9	99	KT327866.1	PH15NI, PH15I
*Lactobacillus helveticus*	7	99	KP764179.1	PH15I
*Zymomonas mobilis*	10	100	CP003715.1	PH15NI
Uncultured bacterium	11, 12, 13, 14	99	LN875309.1	PH15NI, PH15I
*Acetobacter pasteurianus*	15, 16, 17, 18, 19, 20	100	KM983001.1	PH15NI, PH15I
*Acetobacter* sp.	21	98	KC796695.1	PH15NI
*Gluconobacter oxydans*	22, 23, 24	99	CP003926	PH15NI, PH15I
*Bacillus* sp.	25, 26	99	JF309224	PH15NI
**Eukaryotes**				
*Hanseniaspora uvarum*	1, 2	99	KC544511	PH15NI
*Saccharomyces cerevisiae*	3, 5, 6, 7, 8, 9, 10	99	KT229544.1	PH15NI, PH15I
*Saccharomyces* sp.	4	98	KU350335.1	PH15NI
*Theobroma cacao*	11	97	JQ228377.1	PH15NI
*Rhodotorula mucilaginosa*	12, 13	100	HM588765	PH15I
*Lentinula edodes*	14	98	KM015456.1	PH15I
*Pichia kluyveri*	15, 16, 17, 18, 20	99	FM864201	PH15NI, PH15I
*Trichosporon asahii*	19	97	JQ425402	PH15I

^a^ Bands are numbered as indicated on the DGGE gel. PH15NI: PH15 non-inoculated; PH15I: PH15 inoculated.

**Table 2:** Volatile compounds identified by Headspace—Solid Phase Microextraction—Gas Chromatography Mass Spectrometry (HS–SPME GC–MS) during fermentation times (0 h and 144 h) and in chocolate samples, and the reference odor of each compound.

Compounds	Odor Description ^a^	Sample Found
**Acids**		
4-Hydroxybutanoic acid		PH15I Ch
4-Hydroxybutyric acid		PH15NI Ch
Acetic acid	Sour, astringent	PH15NI 144 h
Benzeneacetic acid		PH15NI Ch, PH15I Ch
Butanoic acid	Rancid, butter, cheese	PH15NI Ch, PH15I Ch
Hexanoic acid	Sweat, pungent	All
Isovaleric acid	Sweat, rancid	All
Octanoic acid	Sweaty, fatty	PH15NI (0 h and 144 h), PH15I 144 h, PH15NI Ch
Pentanoic acid		PH15NI Ch, PH15I Ch
Valeric acid	Sweat, acid, rancid	PH15NI Ch, PH15I Ch
**Alcohols**		
2-Ethyl-1-hexanol		PH15NI Ch
1-Hexanol	Fruity, green	PH15NI (0 h and 144 h), PH15I 0 h
1-Methoxy-2-butanol		PH15NI Ch, PH15I Ch
1-Nonanol		PH15NI Ch, PH15I Ch
1-Octanol	Fatty, waxy	All
1-Penten-3-ol		PH15NI 0 h
2,3-Butanediol	Cocoa butter	PH15NI (0 h and 144 h), PH15I 144 h, PH15I Ch
2,4-Pentanediol		PH15NI 0 h, PH15I 0 h, PH15NI Ch, PH15I Ch
2-Furanmethanol		PH15NI Ch
2-Heptanol	Sweet, citrusy	PH15NI (0 h and 144 h), PH15I (0 h and 144 h)
2-Hexanol	Fruity, green	PH15NI 0 h, PH15I 0 h
2-Pentanol	Green, mild green	PH15NI Ch, PH15I Ch
3-Methyl-1-butanol	Malty, chocolate	PH15NI (0 h and 144 h), PH15I (0 h and 144 h)
Benzyl alcohol	Sweet, flower	All
Furfuryl alcohol		PH15I Ch
Guaiacol	Smoke, sweet	PH15NI 144 h, PH15I 144 h
Phenylethyl Alcohol	Honey, rose, caramel	All
**Aldehydes and Ketones**		
3-methylpentanal		PH15NI 0 h, PH15I 0 h, PH15NI Ch, PH15I Ch
(*E*)-2-Heptenal		PH15NI 0 h, PH15I 0 h
(*E*)-2-Nonenal	Tallowy green	PH15NI 0 h, PH15I 0 h
(*E*)-2-Octanal	Fatty, waxy	PH15NI 0 h, PH15I 0 h
(*E*)-2-Undecenal		PH15NI Ch, PH15I Ch
(*E*,*E*)-2,4-heptadienal		PH15NI 0 h, PH15I 0 h, PH15NI Ch, PH15I Ch
1-(2-hydroxyphenyl)ethanone		PH15NI 0 h, PH15I 0 h
2(5*H*)-Furanone	Caramel-like	PH15NI Ch, PH15I Ch
2-Heptanone		PH15NI (0 h and 144 h), PH15I (0 h and 144 h)
2-Hydroxyphenyl methyl ketone		PH15NI 144 h
2-Nonanone		PH15NI 144 h, PH15I (0 h and 144 h)
2-Phenyl-2-butenal	Sweet, roasted	PH15NI 144 h, PH15I 144 h, PH15NI Ch, PH15I Ch
2-Undecenal		PH15NI (0 h and 144 h), PH15I (0 h and 144 h)
3-Methyl-1,2-cyclopentanedione		PH15NI Ch, PH15I Ch
3-Penten-2-one		PH15NI Ch
4-hydroxy-3-methylbutanal		PH15NI 0 h, PH15I 0 h
4-Methylhexanal		PH15NI Ch, PH15I Ch
5-Methyl-2-furaldehyde		PH15NI Ch, PH15I Ch
5-Methyl-2-phenyl-2-hexenal		PH15NI Ch, PH15I Ch
3-methylpentanal		PH15NI 0 h, PH15I 0 h, PH15NI Ch, PH15I Ch
Acetophenone	Floral	PH15NI (0 h and 144 h), PH15I (0 h and 144 h)
Benzaldehyde	Bitter	All
Benzeneacetaldehyde		PH15NI Ch, PH15I Ch
Nonanal		PH15NI 0 h, PH15I 0 h, PH15NI Ch, PH15I Ch
Octanal		PH15NI 0 h, PH15I 0 h, PH15NI Ch, PH15I Ch
Pyranone		PH15NI Ch, PH15I Ch
Acetoin	Butter, cream	PH15NI 144 h, PH15I 144 h, PH15NI Ch, PH15I Ch
**Esters**		
1-methylbutyl benzoate		PH15NI 144 h, PH15I 144 h
1-Methylhexyl acetate		PH15NI 144 h
2-Ethyl-1-hexyl acetate		PH15I Ch
2-Pentanyl benzoate		PH15NI 0 h, PH15I 0 h, PH15NI Ch, PH15I Ch
2-Phenethyl acetate	Fruity	PH15NI 144 h, PH15I 144 h
3-methylbutyl formate		PH15NI 0 h
Amyl acetate	Fruity, banana	PH15NI 144 h, PH15I 144 h
Dibutyl phthalate		PH15NI 144 h, PH15I (0 h and 144 h)
Diisobutyl phthalate		PH15NI Ch, PH15I Ch
Ethyl 2-hydroxypropanoate		PH15NI 144 h, PH15I 144 h
Ethyl benzeneacetate		PH15NI 144 h
Ethyl caprate	Pear, grape	PH15NI 144 h, PH15I 144 h
Ethyl caprylate	Fruity, flowery	PH15I (0 h and 144 h), PH15I Ch
Ethyl laurate	Fruity, floral	PH15NI 144 h, PH15I 144 h, PH15NI Ch, PH15I Ch
Ethyl myristate	Waxy, soapy	PH15NI 144 h, PH15I 144 h
Ethyl palmitate	Waxy, green	PH15NI (0 h and 144 h), PH15I (0 h and 144 h)
Ethyl phenylacetate		PH15NI 0 h
Hexyl acetate		PH15NI 144 h
Isoamylformate		PH15I 0 h
Isobutyl phthalate		PH15NI (0 h and 144 h), PH15I (0 h and 144 h)
Isopropyl palmitate		PH15I 0 h
Methyl Palmitate		PH15NI 144 h, PH15I (0 h and 144 h)
Phenylethyl acetate	fruity, sweet	PH15NI Ch, PH15I Ch
**Pyrazines**		
2,3,5,6-Tetramethylpyrazine	Chocolate, coffee	PH15NI Ch, PH15I Ch
2,3,5-Trimethyl-6-isopentylpyrazine		PH15NI Ch, PH15I Ch
2,3,5-Trimethylpyrazine	Cocoa, rusted nuts	PH15NI Ch, PH15I Ch
2,3-Dimethylpyrazine	Caramel, cocoa	PH15I Ch
2,5-Dimethyl-3-isoamylpyrazine		PH15NI Ch, PH15I Ch
2,5-Dimethylpyrazine	Cocoa, rusted nuts	PH15NI Ch, PH15I Ch
2,6-Dimethylpyrazine	Nutty, coffee, green	PH15I Ch
2-Acetyl-3,5-dimethylpyrazine		PH15NI Ch, PH15I Ch
2-Ethyl-3,6-dimethylpyrazine	Roasted, smoky	PH15NI Ch, PH15I Ch
2-Ethyl-6-methylpyrazine		PH15NI Ch, PH15I Ch
2-Ethylpyrazine	Peanut butter, nutty	PH15NI Ch
2-Methyl-3,5-diethylpyrazine		PH15NI Ch
2-Methyl-6-vinylpyrazine		PH15NI Ch
2-Methylpyrazine	Chocolate, cocoa, nuts	PH15NI Ch, PH15I Ch
**Pyrroles**		
1,3-Dimethyl-5-pyrazolinone		PH15NI Ch, PH15I Ch
2-Acetylpyrrole	Chocolate, hazelnut	PH15NI Ch, PH15I Ch
2-Pyrrolidinone		PH15NI Ch, PH15I Ch
Pyrrole-2-carboxaldehyde		PH15NI Ch, PH15I Ch
**Terpenoids**		
(*E*)-Linalool oxide	Floral, green	PH15NI (0 h and 144 h), PH15I (0 h and 144 h)
(*Z*)-Linalool oxide	Floral	PH15NI 0 h, PH15I 0 h
Linalool	Flower, lavender	PH15NI (0 h and 144 h), PH15I (0 h and 144 h)
**Others ^b^**		
1-methoxy-2-methylpropane		PH15NI (0 h and 144 h), PH15I 144 h
2-Butyltetrahydrofuran		PH15NI Ch, PH15I Ch
2-Pentylfuran		All
7-methyl pentadecane		PH15I (0 h and 144 h)
Hexadecane		PH15I (0 h and 144 h)

^a^ Obtained from literature; ^b^ Includes: furans and alkanes; Abbreviations: Ch: chocolate.

## References

[1] Pereira J.L., Ram A., Figueiredo J.M., Almeida L.C.C. (1990). The first occurrence of “witches’ broom” disease in the principal cocoa growing region of Brazil. Trop. Agric..

[2] Lopes U.V., Monteiro W.R., Pires J.L., Clement D., Yamada M.M., Gramacho K.P. (2011). Cacao breeding in Bahia, Brazil: Strategies and results. Crop Breed. Appl. Biotechnol..

[3] Moreira I.M.V., Miguel M.G.C.P., Duarte W.F., Dias D.R., Schwan R.F. (2013). Microbial succession and the dynamics of metabolites and sugars during the fermentation of three different cocoa (*Theobroma cacao* L.) hybrids. Food Res. Int..

[4] Ramos C.L., Dias D.R., Miguel M.G.C.P., Schwan R.F. (2014). Impact of different cocoa hybrids (*Theobroma cacao* L.) and *S. cerevisiae* UFLA CA11 inoculation on microbial communities and volatile compounds of cocoa fermentation. Food Res. Int..

[5] Clapperton J.F., Lockwood R., Yow S.T.K., Lim D.H.K. (1994). Effects of planting materials on flavour. Cocoa Grow. Bull..

[6] Efraim P., Pires J.L., Garcia A.O., Grimaldi R., Luccas V., Pezoa-Garcia N.H. (2013). Characteristics of cocoa butter and chocolates obtained from cocoa varieties grown in Bahia, Brazil. Eur. Food Res. Technol..

[7] Menezes A.G.T., Batista N.N., Ramos C.L., Silva A.R.A., Efraim P., Pinheiro A.C.M., Schwan R.F. (2016). Investigation of chocolate produced from four different Brazilian varieties of cocoa (*Theobroma cacao* L.) inoculated with *Saccharomyces cerevisiae*. Food Res. Int..

[8] Oliveira B.F., Silva S.D.V.M., Damaceno V.O., Filho L.P.S. (2009). Identificação de fontes de resistência a *Ceratocystis cacaofunesta* em mudas de cacaueiro. Agrotópica.

[9] Pires J.L., Rosa E.S., Macêdo M.M. (2012). Avaliação de clones de cacaueiro na Bahia, Brasil. Agrotópica.

[10] Silva S.D.V.M., Pinto L.R.M., Oliveira B.F., Damaceno V.O., Pires J.L., Dias C.T.S. (2012). Resistência de progênies de cacaueiro à murcha-de-Ceratocystis. Trop. Plant Pathol..

[11] Biehl B., Meyer B., Crone G., Pollmann L., Said M.B. (1989). Chemical and physical changes in the pulp during ripening and post-harvest storage of cocoa pods. J. Sci. Food Agric..

[12] Schwan R.F. (1998). Cocoa fermentations conducted with a defined microbial cocktail inoculum. Appl. Environ. Microbiol..

[13] Afoakwa E.O., Paterson A., Fowler M., Ryan A. (2008). Flavor formation and character in cocoa and chocolate: A critical review. Crit. Rev. Food Sci..

[14] Pereira G.V.M., Miguel M.G.C.P., Ramos C.L., Schwan R.F. (2012). Microbiological and physicochemical characterization of small-scale cocoa fermentations and screening of yeast and bacteria strains to develop a defined starter culture. Appl. Environ. Microb..

[15] Schwan R.F., Pereira G.V.M., Fleet G.H., Schwan R.F., Fleet G.H. (2014). Microbial activities during cocoa fermentation. Cocoa and Coffee Fermentations.

[16] Ardhana M., Fleet G. (2003). The microbial ecology of cocoa bean fermentations in Indonesia. Int. J. Food Microbiol..

[17] Schwan R.F., Wheals A.E. (2004). The microbiology of cocoa fermentation and its role in chocolate quality. Crit. Rev. Food Sci..

[18] Jespersen L., Nielsen D.S., Hønholt S., Jakobsen M. (2005). Occurrence and diversity of yeasts involved in fermentation of West African cocoa beans. FEMS Yeast Res..

[19] Nielsen D.S., Teniola O.D., Ban-Koffi L., Owusu M., Andersson T.S., Holzapfel W.H. (2007). The microbiology of Ghanaian cocoa fermentations analysed using culture dependent and culture independent methods. Int. J. Food Microbiol..

[20] Pereira G.V.M., Magalhães K.T., Almeida E.G., Coelho I.S.C., Schwan R.F. (2013). Spontaneous cocoa bean fermentation carried out in a novel-design stainless steel tank: Influence on the dynamics of microbial populations and physical-chemical properties. Int. J. Food Microbiol..

[21] Camu N., De Winter T., Addo S.K., Takarama J.S., Bernaert H., De Vuyst L. (2008). Fermentation of cocoa beans: Influence of microbial activities and polyphenol concentrations on the flavour of chocolate. J. Sci. Food Agric..

[22] Papalexandratou Z., Vranckena G., De Bruyneb K., Vandammeb P., De Vuys L. (2011). Spontaneous organic cocoa bean box fermentations in Brazil are characterized by a restricted species diversity of lactic acid bacteria and acetic acid bacteria. Food Microbiol..

[23] Schwan R.F., Rose A.H., Board R.G. (1995). Microbial fermentation of cocoa beans, with emphasis on enzymatic degradation of the pulp. J. Appl. Bacteriol..

[24] Rodriguez-Campos J., Escalona-buendía H.B., Orozco-Avila I., Lugo-Cervantes E., Jaramillo-Flores M.E. (2011). Dynamics of volatile and non-volatile compounds in cocoa (*Theobroma cocoa* L.) during fermentation and drying process using principal components analysis. Food Res. Int..

[25] Ho V.T.T., Zhao J., Fleet G. (2014). Yeasts are essential for cocoa bean fermentation. Int. J. Food Microbiol..

[26] Albertini B., Schoubben A., Guarnaccia D., Pinelli F., Vecchia M.D., Ricci M., Renzo G.C.D., Blasi P. (2015). Effect of Fermentation and Drying on Cocoa Polyphenols. J. Agric. Food Chem..

[27] Rodriguez-Campos J., Escalona-Buendía H., Contreras-Ramos S., Orozco-Avila I., Jaramillo-Flores E., Lugo-Cervantes E. (2012). Effect of fermentation time and drying temperature on volatile compounds in cocoa. Food Chem..

[28] Serra-Bonvehí J. (2005). Investigation of aromatic compounds in roasted cocoa powder. Eur. Food Res. Technol..

[29] Aculey P., Snitkjaer P., Owusu M., Bassompiere M., Takrama J., Nørgaard L. (2010). Ghanaian cocoa bean fermentation characterized by spectroscopic and chromatographic methods and chemometrics. J. Food Sci..

[30] Frauendorfer F., Schieberle P. (2008). Changes in key aroma compounds of Criollo cocoa beans during roasting. J. Agric. Food Chem..

[31] Luna F., Crouzillat D., Cirou L., Bucheli P. (2002). Chemical Composition and Flavor of Ecuadorian Cocoa Liquor. J. Agric. Food Chem..

[32] Batista N.N., Ramos C.L., Ribeiro D.D., Pinheiro A.C.M., Schwan R.F. (2015). Dynamic behavior of *Saccharomyces cerevisiae*, *Pichia kluyv*eri and *Hanseniaspora uvarum* during spontaneous and inoculated cocoa fermentations and their effect on sensory characteristics of chocolate. LWT Food Sci. Technol..

[33] Moskowitz H.R. (1983). Product Testing and Sensory Evaluation of Foods: Marketing and R &D Approaches.

[34] Pineau N., Schich P., Cordelle S., Mathonnièrea C., Issanchouc S., Imbertd A., Köster E. (2009). Temporal dominance of Sensations: Construction of the TDS curves and comparison with time intensity. Food Qual. Pref..

[35] Nunes C.A., Pinheiro A.C.M. (2002). SensoMaker Software Version 1.8.

